# Effects of Dietary Protein Levels on Growth Performance, Carcass Traits, Serum Metabolites, and Meat Composition of Tibetan Sheep during the Cold Season on the Qinghai-Tibetan Plateau

**DOI:** 10.3390/ani10050801

**Published:** 2020-05-06

**Authors:** Xungang Wang, Tianwei Xu, Xiaoling Zhang, Yuanyue Geng, Shengping Kang, Shixiao Xu

**Affiliations:** 1 Northwest Institute of Plateau Biology, Chinese Academy of Sciences, Xining 810001, China; wangxg@nwipb.cas.cn (X.W.); xutianwei@nwipb.cas.cn (T.X.); 18362985573@163.com (X.Z.); gengyuanyue19@mails.ucas.ac.cn (Y.G.); kangsp@nwipb.cas.cn (S.K.); 2 University of Chinese Academy of Sciences, Beijing 100049, China

**Keywords:** protein, Tibetan sheep, growth performance, carcass traits, serum metabolites, meat composition

## Abstract

**Simple Summary:**

The Tibetan sheep (*Ovis aries*) is an ovine breed well adapted to the harsh plateau environment. For a long period of time, Tibetan sheep farming has been an important pillar industry for native herders on the Qinghai-Tibetan Plateau. With the increasing demand for sheep meat and products, nutrition research has gained increasing importance. In this study, the effects of three different dietary protein levels (10.1%, 12.1%, and 14.1%) on growth performance, carcass traits, serum metabolites, and meat composition were investigated in Tibetan sheep during the cold season. The results showed that the diets containing 12.1% and 14.1% protein were better than a diet containing 10.1% protein for enhancing the growth performance, carcass performance, and meat products of the Tibetan sheep.

**Abstract:**

Dietary protein is a critical nutrient that directly influences the health and production of livestock. Recent studies showed that protein supplements could enhance the growth performance of Tibetan sheep. However, there is a lack of information regarding the influence of dietary protein levels on carcass traits and meat composition. This study investigated the effects of dietary protein levels on growth performance, carcass traits, serum metabolites, and meat composition in Tibetan sheep during the cold season on the Qinghai-Tibetan Plateau. A total of eighteen 1-year-old, healthy, castrated Tibetan sheep with similar initial body weights (31.7 ± 0.72 kg) were randomly divided into three treatment groups with different dietary protein level (low protein (LP, 10.1%); medium protein (MP, 12.1%); high protein (HP, 14.1%)) diets. The results indicated that the Tibetan sheep fed with the MP and HP diets had greater final body weights (BWs), average daily gains (ADGs), and average daily feed intakes (ADFIs) (*p* < 0.05). The MP and HP diets also improved the hot carcass weight, net meat (including the fat) weight, and bone weight of the sheep significantly (*p* < 0.05). Besides, the dietary protein levels could significantly affect the serum concentrations of growth hormone (*p* < 0.05). The diameter of muscle fibers in the MP group was significantly greater than that in the LP group (*p* < 0.05), while the density of muscle fibers showed the opposite trend. The dietary protein levels only significantly (*p* < 0.05) influenced the ether extract content and profile content of the longissimus dorsi muscle but had no effect on other parameters of meat composition. In summary, our results indicate that dietary protein levels affect growth performance, carcass traits, and meat composition and that diets containing 12.1% and 14.1% protein are recommended to obtain better production performance and meat products in Tibetan sheep, rather than a diet containing 10.1% protein, during the cold season on the Qinghai-Tibetan Plateau.

## 1. Introduction

The Qinghai-Tibetan Plateau (QTP) is a special ecosystem with an extreme environment, including a high altitude, a low temperature, and strong UV radiation [1,2]. Tibetan sheep (*Ovis aries*) are an important livestock resource, raised at an altitude above 3000 m on the QTP [3]. At present, there are more than fifty million Tibetan sheep well adapted to the harsh environment and that provide meat and other economic production for about five million native Tibetan herders [4,5,6]. Based on traditional livestock management, Tibetan sheep graze on the natural grazing lands all year-round [7]. However, during the long periods of cold season, there is a desperate shortage of grassland productivity and herbage nutrition, and Tibetan sheep often lose live-weight [8,9]. In recent years, a new production system is being adopted on the QTP. During the warm season (June to October, average temperature approximately −2 to 12 °C) sheep are largely dependent on natural grazing lands, but in the cold season (November to the following May, average temperature from approximately −5 to −15 °C), the sheep are housed and fed in sheds to ensure normal growth [10]. Under this novel production system, the growth and development of sheep can be improved effectively. Therefore, a better understanding of how to efficiently feed sheep would be beneficial for livestock production on the QTP.

As an important nutrient in diets, protein is a crucial for livestock’s nutrient digestibility and production performance [11]. Previous studies showed that feeding protein supplements could significantly promote growth performance, feed efficiency, and economic benefits in Tibetan sheep [12]. Furthermore, several studies reported that supplementing Tibetan sheep during the cold season could also increase rumen microbial growth and improve rumen epithelium development and absorptive capability [7,13]. However, these studies focused on the growth performance and rumen function of sheep, with less attention to carcass traits, serum metabolites, muscle fiber characteristics, and meat composition. Hence, the objective of this study was to evaluate the effects of feeding different levels of dietary protein to Tibetan sheep on their growth performance, serum metabolites, carcass traits, and meat composition during the cold season on the QTP.

## 2. Materials and Methods

### 2.1. Ethics Statement

The animal procedures in this study were performed according to the Guideline for the Care and Use of Laboratory Animals in China. This study was approved by the Experimental Animal Use Ethics Committee of the Northwest Institute of Plateau Biology, CAS (Permit No. NWIPB20160302).

### 2.2. Experimental Design

Eighteen 1-year-old, healthy, castrated Tibetan sheep with similar initial body weights (BWs) of 31.7 ± 0.72 kg were selected from Haibei Demonstration Zone of Plateau Modern Ecological Husbandry Science and Technology in Qinghai Province (China) for the feeding trial. The sheep were randomly divided into three dietary treatment groups, with each group containing 6 sheep. The protein levels of the three diets were 10.1% (low protein, LP), 12.1% (medium protein, MP), 14.1% (high protein, HP). The diets were designed according to the National Research Council [14] and were isoenergetic (10.1 MJ metabolizable energy (ME)/kg). The ingredients and nutrient compositions of three diets are shown in Table 1 and the analysis of nutrient composition in the diets was performed according to the Association of Official Analytical Chemists (AOAC) procedures [15]. All diets had the same forage to concentrate ratio of 50:50. The concentrate and oat hay were manually mixed and fed (dry matter basis) at 3.5% BW of sheep [16]. The sheep were fed twice daily at 8:00 and 17:00 with the same amount, and each sheep was maintained in an individual pen. Fresh water was provided ad libitum throughout the experiment. The whole formal experiment took place over 105 d after 15 d of adaption to the experimental diets. Sheep were weighted on d 0, d 35, d 70, and d 105 before feeding in the morning to calculate the average daily gain (ADG), and feed intake was recorded each day to calculate the average daily feed intake (ADFI) and feed/gain ratio (F/G).

### 2.3. Carcass Traits

At the end of the experiment, four sheep from each group were selected randomly and transported to a commercial abattoir located 16 km away from the experimental site. Sheep were fasted for 24 h before slaughter. At the commercial abattoir, the sheep were stunned and slaughtered humanely according to standard commercial procedures (NY 467-2001, The Ministry of Agriculture of the People’s Republic of China). The hot carcass weight, net meat (including the fat) weight, and bone weight were recorded and used to calculate the carcass traits. These parameters were calculated as follows: % carcass yield = hot carcass weight/live weight × 100; % net meat (including the fat) yield = net meat (including the fat) weight/live weight × 100; bone/meat = bone weight/net meat (including the fat) weight.

### 2.4. Serum Metabolites

At the end of the experiment, 10 mL of blood was collected from the jugular vein of each sheep before feeding in the morning, and the serum was separated by centrifugation at 2000× *g* for 10 min at 4 °C. The concentration of serum growth hormone (GH) was determined by the sheep GH ELISA kit (CV% < 15%, CUSABIO Biotech Co. Ltd., Wuhan, China) according to the instruction manual, and the levels of total protein (TP), albumin (ALB), glucose (GLU), globulin (GLO), total cholesterol (TCH), triglyceride (TG), glutamic-pyruvic transaminase (ALT), and glutamic oxaloacetic transaminase (AST) in the serum were determined by using the Catalyst One Biochemical Analyzer (Idexx Laboratories, Westbrook, ME, USA).

### 2.5. Muscle Fiber Characteristics

After slaughter, samples of longissimus dorsi muscle adjacent to the 13th thoracic vertebra (approximately 1 cm^3^) from each sheep were collected and fixed in tissue fixation fluid overnight. After dehydration in a series of ethanol gradient solutions (70%, 80%, 90%, and then 95% anhydrous alcohol for 2 min at each solution) and inducing transparency with xylene, samples were embedded in paraffin and were sectioned in serial transverse sections (5 μm thick). Dewaxed serial sections were stained with the traditional hematoxylin and eosin (HE) method to show the histological characteristics of the muscle fibers [17]. The morphological characteristics of the muscle fibers (e.g., diameter, perimeter, area, and density) were measured using CaseViewer 2.2 (3DHISTECH Ltd., Budapest, Hungary) under an electron microscope (Axio IMAGE Z2, Leica microsystems Ltd., Wetzlar, Germany).

### 2.6. Meat Composition

At the time of slaughter, samples of longissimus dorsi muscle between the 9th and 11th ribs (approximately 200 g) were collected from each sheep to measure nutrient contents. Dry matter, crude protein, ether extract, and crude ash contents were assayed according to the AOAC procedures [15]. The concentrations of 17 common amino acids were determined with an automatic amino acid analyzer (S433D, Sykam Ltd., Munich, Germany) according to the methods described by Peterson et al. [18].

### 2.7. Statistical Analyses

All the data in this study are presented as the mean values and the standard errors of the mean (SEM), and analyzed by using one-way ANOVA and Tukey post hoc tests in IBM SPSS 22.0 (SPSS Inc., Chicago, IL, USA). In this study, the diet was treated as the main effect, and the sheep was the experimental unit. Significance was declared at *p* < 0.05.

## 3. Results

### 3.1. Growth Performance

The effects of dietary protein levels on the BW, ADG, ADFI, and F/G of Tibetan sheep are shown in Table 2. At the first feeding stage (1–35 d), the dietary protein levels did not significantly (*p* > 0.05) affect the final BW, ADG, and F/G of the Tibetan sheep. During the second feeding stage (36–70 d), the final BW in the LP group was significantly lower than that in the MP group (*p* < 0.05). The ADG and F/G were significantly different between groups commencing during the last feeding stage (71–105 d), and the LP group had the lowest ADG and the greatest value of F/G (*p* < 0.05). During the whole experiment (1–105 d), the LP group had the lowest final BW, ADG, and ADFI and the greatest value of F/G (*p* < 0.05).

### 3.2. Carcass Traits

The effects of dietary protein levels on carcass traits are shown in Table 3. The live weight, hot carcass weight, net meat (including the fat) weight, and bone weight in the LP group were significantly lower than those in the MP and HP groups (*p* < 0.05). The HP group had the greatest value for the bone/meat ratio value (*p* < 0.05). By contrast, the dietary protein levels did not significantly (*p* > 0.05) influence the carcass yield and net meat (including the fat) yield of the groups of Tibetan sheep.

### 3.3. Serum Metabolites

The effects of dietary protein levels on serum metabolites are shown in Table 4. The level of GH in the LP group was significantly lower than that in the MP and HP groups (*p* < 0.05). Nevertheless, dietary protein levels had no significant effect on serum TP, ALB, GLU, GLO, TCH, TG, ALT, and AST content (*p* > 0.05).

### 3.4. Muscle Fiber Characteristics

**Figure 1:**
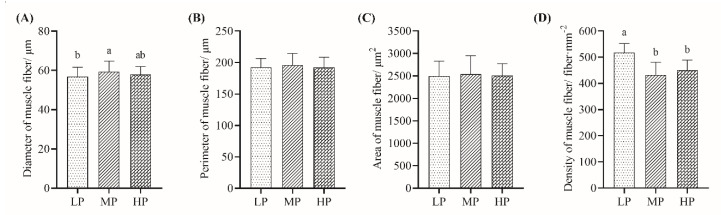
Effects of the three different dietary protein levels on the histological characteristics of longissimus dorsi muscle in Tibetan sheep. (**A**) Diameter of muscle fibers. (**B**) Perimeter of muscle fibers. (**C**) Area of muscle fibers. (**D**) Density of muscle fibers. ^a, b^ Indicate significant differences among three groups.

As shown in Figure 1, the diameter of muscle fibers in the LP group was significantly lower than that in the MP group (*p* < 0.05), while no significant difference was detected between the MP and HP groups (*p* > 0.05) or between the LP and HP groups (*p* > 0.05). For the density of muscle fibers, the greatest value was detected in the LP group. Dietary protein levels did not significantly (*p* > 0.05) influence the perimeter and area of muscle fibers.

### 3.5. Nutrient Contents of the Longissimus Dorsi Muscle

The ether extract was significantly lower in the longissimus dorsi muscle of sheep in the LP group than in the MP group (*p* < 0.05) (Table 5). Dietary protein levels had no significant influence on the dry matter, crude protein, and crude ash contents of longissimus dorsi muscle (*p* > 0.05).

### 3.6. Amino Acid Profile of Longissimus Dorsi Muscle

As shown in Table 6, eight essential amino acids (EAA) and nine nonessential amino acids (NEAA) in the longissimus dorsi muscle of Tibetan sheep were detected by an automatic amino acid analyzer. The content of proline was significantly lower in the LP group than that in the MP group (*p* < 0.05), but no differences between groups were observed in other amino acids.

## 4. Discussion

### 4.1. Growth Performance

Recent studies had showed that dietary nutrient level was closely related to the growth performance of livestock [19,20,21]. Besides, it is reported that the proportion of concentrate to forage could also affect the BW, ADG, and F/G of calves and Tibetan sheep [16,22,23]. In the present study, the dietary protein levels did not significantly influence the BW and ADG of Tibetan sheep during the first feeding stage (1–35 d). During the whole experiment (1–105 d), the greater final BW, ADG, and ADFI observed in the MP and HP groups indicates that these diets are optimal for achieving the best growth performance of Tibetan sheep during the cold season on the QTP. Furthermore, the LP group had a greater value of F/G than other two groups, indicating that the LP diet had the lowest feed conversion efficiency of these three diets. Similar findings were observed by Estrada-Angulo et al. [24], and it was reported that increasing the dietary protein level could increase the ADG and gain efficiency of Pelibuey × Katahdin lambs. Karlsson and Martinsson [11] also found that the lambs fed with high protein supplements had a greater total gain and ADG and more efficient feed conversion. Based on these previous reports and our findings, we speculate that an appropriate dietary protein level could enhance protein availability and increase amino acid absorption in animals and eventually result in increased growth performance [25,26].

### 4.2. Carcass Traits

In recent years, carcass traits are crucial indices reflecting livestock production in animal husbandry [27,28,29]. Francisco et al. [30] showed that the live BW and diet composition were two main factors associated with carcass traits, and these observations were also found in our study. As the LP diet contained lower levels of nutrients than other two diets, the sheep in the LP group exhibited poorer growth performance, as shown by the lower hot carcass weight, net meat (including the fat) weight, and bone weight. Furthermore, Cortese et al. [31] reported that the carcass yield of Charolais bulls was not affected by increased dietary protein levels. Luo et al. [28] also showed that there were no differences in backfat thickness and carcass yield in fattening pigs fed with different levels of *β*-glucan supplementation. The above results were consistent with the results of this study. Although the hot carcass weight changed with the dietary protein levels, the carcass yield and net meat (including the fat) yield were independent of the respective dietary protein.

### 4.3. Serum Metabolites

Serum metabolites are important biochemical parameters reflecting the health status of an animal and its metabolic activity [32]. In recent years, serum biomarkers have been used as diagnostic standards for assessing animal health due to their convenient and economical features [7,33,34]. Recent research had reported that greater GH levels enhance lipid mobilization in animals, which is essential in promoting muscle and bone growth [35,36]. Our study found that the GH levels in sheep in the LP group were significantly lower than in sheep in the MP and HP groups. The results for the serum concentrations of TP, ALB, GLU, GLO, TCH, TG, ALT, and AST from the sheep in all three groups were within the normal physiological range, but no differences among treatments were detected. This is consistent with the previous findings that dietary concentrate levels had no significant effect on most blood parameters in Holstein heifers [37]. Our results also showed that providing the Tibetan sheep with adequate nutrition under the cold season could ensure their normal metabolism and physiological functions.

### 4.4. Muscle Fiber Characteristics

Muscle fiber structure is a quantitative index to evaluate the meat composition at the cellular level [38]. In the present study, we found that the histological characteristics of the longissimus dorsi muscle fibers in Tibetan sheep could significantly affected by the dietary protein levels, and the diameter of muscle fibers was negatively correlated with density. Şirin et al. [39] reported that there was a strong relationship between muscle fiber composition and the growth performance of lambs. Fahey et al. [40] showed that the diet composition and nutritional status could alter the muscle fiber development of newborn lambs. Furthermore, the effects of a high nutritional level may involve an increase in and prolongation of myoblast proliferation [41]. In our study, the sheep fed the LP diet had the lowest diameters of muscle fibers, possibly due to poorer growth performance and lower dietary nutrition intake. Unfortunately, observations regarding the different types of muscle fiber (e.g., Type I, Type IIA, and Type IIB) were not recorded in the present study, which would help to understand whether dietary protein levels had any deeper effects on the muscle fibers of Tibetan sheep.

### 4.5. Meat Composition

Previous studies have indicated that dietary nutrients play a pivotal role in regulating the meat composition of livestock. For instance, increased dietary energy increased the content of intramuscular fat of Holstein-Friesians bulls [42]. Moreover, dietary supplementation with sea buckthorn pomace increased the crude fat content of lambs, but had no significant influence on other nutrient contents [43]. In the present study, we found that dietary protein levels significantly affect the ether extract content of Tibetan sheep, and the MP group had the greatest value. A previous study reported that diet composition, particularly the protein/energy ratio, could influence fatness [44]. In our study, the MP and HP diets had higher abundances of protein components (e.g., soybean meal and rapeseed meal) and contributed to the greater ADFI of Tibetan sheep. In this sense, the greater ether extract content may also be associated with the elevated nutrient intake. Yin et al. [45] reported that the composition and content of amino acids are critical evaluation indices, which are associated with the flavor and nutritional value of meat. Our results showed that the meat of Tibetan sheep was rich in a variety of essential and nonessential amino acids like other livestock (e.g., Hu sheep, the Hengshan goat, and Yanbian cattle) distributed in the low-altitude areas of China [46,47,48]. It indicated that Tibetan sheep meat appears to be an excellent quality protein food source for native residents living at altitudes greater than 3000 m on the QTP. Besides, these results showed that the content of amino acids in longissimus dorsi muscle may not be directly related to dietary protein levels, and the specific reasons for this still need to be explored in further depth.

## 5. Conclusions

Based on the results, Tibetan sheep fed with the MP diet (containing 12.1% protein) and HP diet (containing 14.1% protein) had better growth performance, associated with elevated serum GH but no differences in other serum biochemical parameters. Furthermore, dietary protein levels could affect the histological characteristics of the longissimus dorsi muscle in Tibetan sheep. The results suggest that changes in the diet composition may affect the ether extract content of the longissimus dorsi muscle by altering the food intake of the Tibetan sheep. This study could facilitate applications in Tibetan sheep production during the cold season on the QTP.

## Figures and Tables

**Table 1:** Ingredients and nutrient levels of the experimental diets with three different protein levels (on a dry matter basis).

Item	Diet ^1^		
	LP	MP	HP
Ingredients, g/kg			
Oat hay	500	500	500
Corn grain	210	165	120
Wheat grain	135	120	105
Wheat bran	70	75	80
Soybean meal	35	55	75
Rapeseed meal	25	60	95
NaCl	5	5	5
CaHPO_4_·2H_2_O	3	3	3
Bentonite	5	5	5
CaCo_3_	4.5	4.5	4.5
NaHCO_3_	2.5	2.5	2.5
Premix ^2^	5	5	5
Nutrient levels ^3^			
CP (%)	10.1	12.1	14.1
ME (MJ/kg)	10.1	10.1	10.1
EE (%)	2.7	2.8	2.9
NDF (%)	37.5	38.5	39.5
ADF (%)	19.1	20.1	21.1
Ca (%)	0.6	0.7	0.7
P (%)	0.4	0.5	0.5

^1^ LP: low protein; MP: medium protein; HP: high protein. ^2^ Premix provided per kg of feed: Vitamin A, 50,000 IU; Vitamin D3, 12,500 IU; Vitamin E, 1000 IU; Cu, 250 mg; Fe, 12,000 mg; Zn, 1000 mg; Mn, 1000 mg; and Se, 7.5 mg. ^3^ ME: metabolizable energy = total digestible nutrients × 0.04409 × 0.82, according the National Research Council [14]; CP: crude protein; EE: ether extract; NDF: neutral detergent fiber; ADF: acid detergent fiber.

**Table 2:** Effects of three different dietary protein levels on growth performance in Tibetan sheep (n = 6 per group).

Parameter ^1^	Treatments			SEM ^2^	*p*-Value
	LP	MP	HP		
1–35 d					
Initial BW, kg	31.6	32.1	31.3	0.17	0.123
Final BW, kg	36.3	38.1	37.2	0.42	0.221
ADG, g/d	133.8	169.8	167.9	13.26	0.488
ADFI, g/d	797 ^c^	926 ^a^	870 ^b^	0.01	0.000
F/G	5.99	5.55	5.24	0.33	0.669
36–70 d					
Initial BW, kg	36.3	38.1	37.2	0.42	0.221
Final BW, kg	41.4 ^b^	45.3 ^a^	42.7 ^ab^	0.58	0.010
ADG, g/d	144.8	204.5	155.5	13.12	0.139
ADFI, g/d	1268 ^b^	1465 ^a^	1289 ^b^	0.03	0.001
F/G	8.74	7.28	8.30	0.27	0.063
71–105 d					
Initial BW, kg	41.4 ^b^	45.3 ^a^	42.7 ^ab^	0.58	0.010
Final BW, kg	47.8 ^b^	55.7 ^a^	52.8 ^a^	1.00	0.001
ADG, g/d	184.3 ^b^	297.1 ^a^	291.0 ^a^	17.39	0.004
ADFI, g/d	1518 ^b^	1880 ^a^	1783 ^a^	0.04	0.000
F/G	8.39 ^a^	6.43 ^b^	6.19 ^b^	0.35	0.011
1–105 d					
Initial BW, kg	31.6	32.1	31.3	0.17	0.123
Final BW, kg	47.8 ^b^	55.7 ^a^	52.8 ^a^	1.00	0.001
ADG, g/d	154.3 ^b^	223.8 ^a^	204.8 ^a^	9.51	0.002
ADFI, g/d	1195 ^c^	1424 ^a^	1314 ^b^	23.13	0.000
F/G	7.87 ^a^	6.33 ^b^	6.53 ^b^	0.27	0.033

Values are expressed as means. ^1^ BW, body weight; ADG, average daily gain; ADFI, average daily feed intake; F/G, feed/gain ratio. ^2^ SEM = standard error of the mean. ^a, b, c^ Values within a row with different superscripts differ significantly at *p* < 0.05.

**Table 3:** Effects of three different dietary protein levels on carcass traits in Tibetan sheep (n = 4 per group).

Parameter	Treatments			SEM ^1^	*p*-Value
	LP	MP	HP		
Live weight, kg	47.8 ^b^	54.0 ^a^	53.5 ^a^	0.993	0.002
Hot carcass weight, kg	22.20 ^b^	26.91 ^a^	26.19 ^a^	0.747	0.005
Carcass yield, %	46.48	49.82	48.97	0.732	0.156
Net meat (including the fat) weight, kg	17.41 ^b^	21.05 ^a^	19.95 ^a^	0.568	0.009
Net meat (including the fat) yield, %	36.46	38.96	37.31	0.588	0.224
Bone weight, kg	4.79 ^b^	5.86 ^a^	6.24 ^a^	0.205	0.001
Bone/meat	0.27 ^b^	0.28 ^b^	0.31 ^a^	0.006	0.001

Values are expressed as means. ^1^ SEM = standard error of the mean. ^a, b^ Values within a row with different superscripts differ significantly at *p* < 0.05.

**Table 4:** Effects of three different dietary protein levels on serum metabolites in Tibetan sheep (n = 6 per group).

Parameter	Treatments			SEM ^1^	*p*-Value
	LP	MP	HP		
Growth hormone (GH), ng/mL	32.5 ^b^	36.1 ^a^	35.5 ^a^	2.05	0.001
Total protein (TP), g/L	69.0	69.2	70.9	2.55	0.136
Albumin (ALB), g/L	35.4	36.4	36.1	1.63	0.342
Glucose (GLU), mmol/L	4.68	4.82	4.81	0.35	0.561
Globulin (GLO), g/L	33.6	32.8	34.8	2.97	0.236
Total cholesterol (TCH), mmol/L	1.46	1.67	1.66	0.29	0.140
Triglyceride (TG), mmol/L	0.29	0.32	0.30	0.08	0.664
Glutamic-pyruvic transaminase (ALT), U/L	19.6	17.2	19.3	5.02	0.458
Glutamic-oxalacetic transaminase (AST), U/L	108.8	115.2	102.7	22.85	0.420

Values are expressed as means. ^1^ SEM = standard error of the mean. ^a, b^ Values within a row with different superscripts differ significantly at *p* < 0.05.

**Table 5:** Effects of three different dietary protein levels on the nutrient contents of the longissimus dorsi muscle in Tibetan sheep (n = 4 per group).

Parameter	Treatments			SEM ^1^	*p*-Value
	LP	MP	HP		
Dry matter, %	24.09	26.39	24.86	0.465	0.113
Crude protein, %	18.63	19.38	19.27	0.205	0.287
Ether extract, %	2.48 ^b^	3.43 ^a^	2.68 ^ab^	0.169	0.033
Crude ash, %	1.02	1.09	0.99	0.024	0.236

Values are expressed as means. ^1^ SEM = standard error of the mean. ^a, b^ Values within a row with different superscripts differ significantly at *p* < 0.05.

**Table 6:** Effects of three different dietary protein levels on the amino acid profile of longissimus dorsi muscle in Tibetan sheep (n = 4 per group).

Amino Acid ^1^	Treatments			SEM ^2^	*p*-Value
	LP	MP	HP		
Threonine, %	3.89	3.77	4.03	0.05	0.101
Valine, %	4.26	4.13	4.19	0.04	0.495
Methionine, %	2.15	2.21	2.22	0.04	0.821
Leucine, %	4.13	4.02	4.15	0.05	0.582
Leucine, %	6.96	6.83	7.16	0.09	0.325
Phenylalanine, %	3.51	3.46	4.03	0.14	0.208
Histidine, %	4.20	3.88	3.84	0.09	0.197
Lysine, %	7.29	7.55	7.58	0.09	0.393
Asparticacid, %	7.80	7.64	8.00	0.09	0.284
Serine, %	3.29	3.18	3.44	0.05	0.050
Glutamicacid, %	14.77	14.22	15.00	0.19	0.235
Glycine, %	3.51	3.44	3.62	0.05	0.255
Alanine, %	4.82	4.70	4.90	0.06	0.365
Cystine, %	0.23	0.18	0.16	0.02	0.236
Tyrosine, %	3.04	2.91	3.40	0.12	0.249
Arginine, %	5.14	4.92	5.32	0.08	0.159
Proline, %	2.75 ^bc^	2.73 ^b^	2.98 ^a^	0.04	0.004
EAA, %	36.38	35.85	37.20	0.31	0.218
NEAA, %	45.36	43.90	46.82	0.56	0.091
TAA, %	78.99	77.02	81.04	0.82	0.134

Values are expressed as means. ^1^ EAA, essential amino acids; NEAA, nonessential amino acids; TAA, total amino acids. ^2^ SEM = standard error of the mean. ^a, b, c^ Values within a row with different superscripts differ significantly at *p* < 0.05.
